# Enhanced detection sensitivity of neuronal activity patterns using CaMPARI1 vs. CaMPARI2

**DOI:** 10.3389/fnins.2022.1055554

**Published:** 2023-01-10

**Authors:** Aniruddha Das, Daniel Margevicius, Julie Borovicka, Jacob Icardi, Davina Patel, Marie-Eve Paquet, Hod Dana

**Affiliations:** ^1^Department of Neurosciences, Lerner Research Institute, Cleveland Clinic Foundation, Cleveland, OH, United States; ^2^Department of Biochemistry, Microbiology and Bioinformatics, CERVO Brain Research Centre, Université Laval, Québec, QC, Canada; ^3^Department of Molecular Medicine, School of Medicine, Cleveland Clinic Lerner College of Medicine, Case Western Reserve University, Cleveland, OH, United States

**Keywords:** calcium sensor, CaMPARI, neurons, activity recording, two-photon microscopy (2-PM)

## Abstract

Calcium-modulated photoactivatable ratiometric integrator (CaMPARI) is a calcium ion (Ca^2+^)- and light-dependent genetically encoded fluorescent activity integrator that can capture snapshots of neuronal activity through an irreversible process known as photoconversion. This unique property was previously used to label neurons based upon their tuning properties in order to map synaptic connectivity and to record large-scale neuronal activity in freely moving mice without attaching any mechanical device to them. The latest version of CaMPARI (CaMPARI2) was engineered to enhance the contrast generated by photoconverting the green protein to the activity-dependent red form and to reduce the Ca^2+^-independent photoconversion rate compared to the first generation of CaMPARI (CaMPARI1). However, here we show that this optimization process also resulted in reduced photoconversion efficiency of active neurons in the mouse cortex and hippocampus. Through side-by-side comparison of the two CaMPARI sensors under several experimental conditions, we show that CaMPARI1 exhibits a substantially higher red-to-green ratio in active cells than CaMPARI2. In addition, we show that CaMPARI1 also functions as a more sensitive traditional Ca^2+^ sensor than CaMPARI2 by producing larger activity-driven dynamic fluorescence changes in the observed neurons. Therefore, we conclude that during the optimization process of CaMPARI2, some of the sensor’s characteristics were not predicted properly by *in vitro* screening assays, and therefore *in vivo* screening and validation steps should be included in future optimization attempts to increase the predictability of screening pipelines.

## Introduction

Over the past two decades, extensive efforts have been invested in designing and optimizing protein-based sensors to record from neurons inside the living brain. The development of genetically encoded calcium indicators (GECIs) especially benefitted from these efforts, with the engineering of multiple GECI variants excited by different wavelengths and utilizing different mechanisms to translate changes in intracellular Ca^2+^ concentration into fluorescence signal (Looger and Griesbeck, 2012; Rodriguez et al., 2017; Grienberger et al., 2022). For example, the ongoing optimization of the GCaMP sensors (Nakai et al., 2001; Tian et al., 2009; Akerboom et al., 2012; Chen et al., 2013; Dana et al., 2019; Inoue et al., 2019; Zhang et al., 2021) enabled the extension of earlier *in vitro* experiments into *in vivo* recordings under multiple experimental conditions and using various animal models. Notably, most of the optimization relied upon *ex vivo* and *in vitro* assays for screening, such as purified protein solutions, bacteria cells, cell lines, and/or cultured mammalian neurons. Usually, the results were validated in one or more *in vivo* models (Tian et al., 2009; Akerboom et al., 2012, 2013; Thestrup et al., 2014; Inoue et al., 2015, 2019; Dana et al., 2016, 2019; Zhang et al., 2021). However, due to the extensive differences between the simplified *in vitro* assays and the more complex *in vivo* environment, the predictive power of this approach may be limited.

Calcium-modulated photoactivatable ratiometric integrator (CaMPARI) is a new type of GECI, which changes its fluorescence emission color from green to red when illuminated with 400 nm light and in an environment with high Ca^2+^ concentration in a process known as photoconversion (PC) (Fosque et al., 2015). In addition, CaMPARI can be used to record neuronal activity in a similar manner to other GECIs by monitoring dynamic changes in its fluorescence intensity. Notably, unlike most GECIs, CaMPARI’s fluorescence becomes dimmer when intracellular Ca^2+^ concentration increases. Previous works in rodents with CaMPARI used its PC capabilities to perform experiments like mapping of functional connectivity (Zolnik et al., 2017), identifying neuron-astrocyte circuitry (Serra et al., 2022), and recording of large-scale, volumetric neuronal activity from freely moving mice without any mechanical restriction (Das et al., 2022). Recently, a new generation of CaMPARI, CaMPARI2, was developed with enhanced PC rate and brighter red fluorescence compared to the first CaMPARI generation (CaMPARI1). Similar to the optimization pipeline described above, CaMPARI2’s optimization process included screening in bacterial lysates and purified protein assays, and then testing in cultured mammalian neurons *in vitro* and validation *in vivo* in mice and zebrafish (Moeyaert et al., 2018). In this work, we show that the development of CaMPARI2 yielded an unexpected decrease in performance *in vivo*. We conducted side-by-side comparisons of the sensitivity of CaMPARI1 and CaMPARI2 to detect visual-evoked activity in the mouse primary visual cortex (V1) and spontaneous activity in hippocampal CA1 neurons using two excitation wavelengths. Although CaMPARI2’s baseline PC rate was lower than CaMPARI1 as predicted by *in vitro* work, its PC rate in active neurons was substantially lower, leading to no improvement in CaMPARI2’s ability to distinguish between stimulated and non-stimulated brain regions. Therefore, we suggest that future optimization processes of neuronal activity sensors will include additional *in vivo* assays to increase the predictability of the screening platform.

## Materials and methods

All experimental and surgical procedures were performed following the set guidelines and protocols approved by the Lerner Research Institute Animal Care and Use Committee (IACUC) and Institutional Biosafety Committee (IBC). Mice were group-housed in standard vivarium conditions until the start of the study. The vivarium was maintained at 20–21°C and food (Teklad 2,918 regular diet, Envigo) and water were available *ad libitum.* Lights were kept on a 12-h light/12-h dark cycle, and experiments were conducted during the light cycle.

### Surgical procedure and virus injection

**FIGURE 1:**
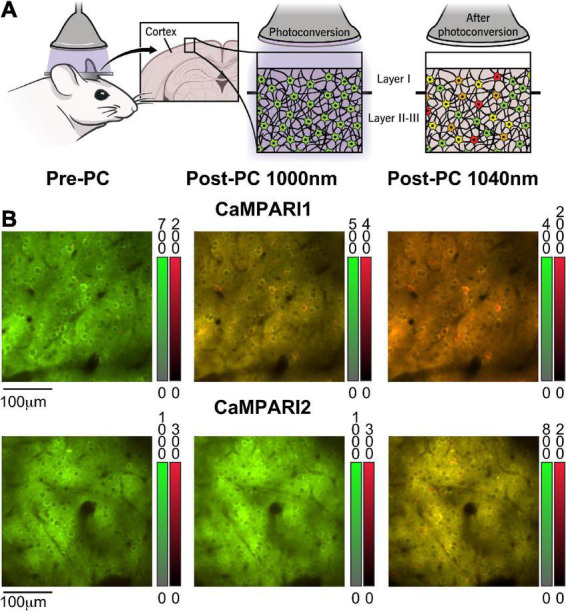
Calcium-modulated photoactivatable ratiometric integrator can measure neuronal activity using either PC or dynamic recording. **(A)** Schematic illustration of CaMPARI’s photoconversion process, as used in this study. A mouse was implanted with a cranial window, injected with an AAV to express CaMPARI, and then illuminated with 400 nm light while presented with a visual stimulus to the contralateral eye (left). Before PC, CaMPARI emits green fluorescence, and when illuminated with 400 nm light it gradually changes its color to red in active neurons (middle and right panels). The transition from green to red is irreversible at the single protein level, and the red signal slowly decays within days as the red protein deteriorates (Das et al., 2022). **(B)** Example images from the mouse V1 *in vivo*, showing neurons expressing CaMPARI1 and CaMPARI2 (upper and lower rows, respectively) before PC (left column), and after PC when captured with 1,000 nm excitation (middle column) or 1,040 nm excitation (right column). Color bars next to each image show the green and red fluorescence signal ranges.

**FIGURE 2:**
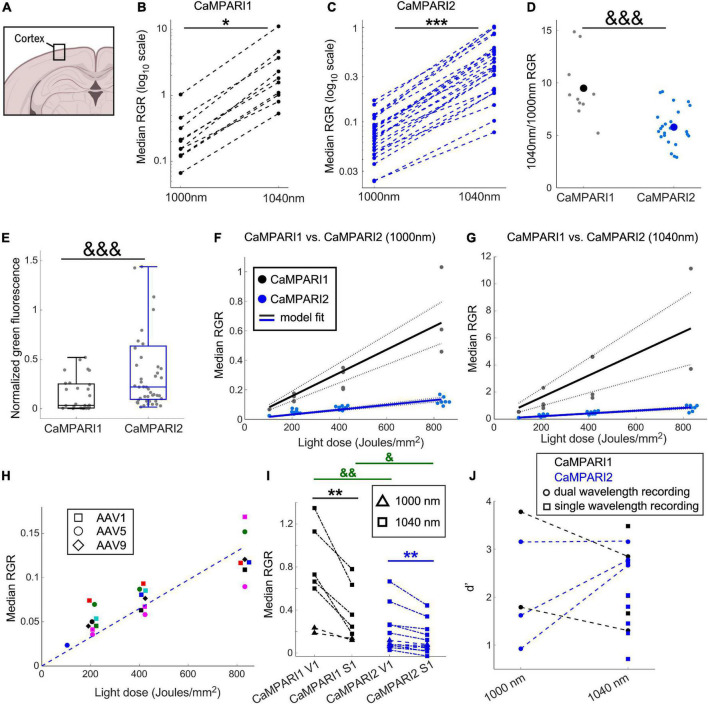
Comparing the PC performance of CaMPARI1 and CaMPARI2 in the mouse V1. **(A)** Schematic illustration of cortical recording. **(B,C)** Comparing the median RGR for CaMPARI1 **(B)** and CaMPARI2 **(C)** shows the increased RGR with longer wavelength excitation. RGR was recorded from all identified neurons in V1, and each dot is a median across all cells from the same mouse on a single recording data were acquired from 4 CaMPARI1 mice, each mouse recorded with 2–3 different light doses (*n* = 443, 234, 185, and 571 cells from each mouse), and 11 CaMPARI2 mice, each mouse recorded with 1–3 different light doses (minimal and maximal numbers of cells/mice were 145 and 1,504, respectively; median value was 526). The RGR readout was performed sequentially for each mouse using 1,000 nm and then 1,040 nm excitation wavelengths. Lines connect the data points acquired from the same mouse during the same session. Note the logarithmic scale of the y-axis. **(D)** The ratios of median RGR values acquired from the same mice at 1,040 and 1,000 nm excitation wavelengths were significantly higher for CaMPARI1 than CAMPARI2 mice [9.05 ± 3.06 vs. 5.8 ± 1.75, respectively, mean ± std.; *p* = 0.0009, Wilcoxon Ranksum Test; data from the same mice as in **(B,C)]**. Small circles show the ratio for median from individual recordings and the large circles are the mean across all data points. **(E)** The median somatic green fluorescence from all neurons within a single FOV before PC (normalized to the square of the excitation power at 1,000 nm) was significantly higher for CaMPARI2 than CaMPARI1, in agreement with the previously published *in vitro* characterization of the CaMPARI constructs (Moeyaert et al., 2018) (data from *n* = 28 and 48 fields of view located 100–150 μm under the pia of 5 and 7 CaMPARI1- and CaMPARI2-expressing mice, respectively; 2–82 cells/field of view, with a median number of 27; *p* = 0.0003, Wilcoxon Rank Sum Test). Each box shows the respective 25–75 percentile of this distribution, horizontal lines show the median values, and the whiskers span the distance from the boxes to the extreme data points. **(F)** The median RGR across all recorded cells showed linear increases with the light dose used for PC, with substantially higher PC rates for CaMPARI1 than CaMPARI2. The individual data points (gray and blue circles for CaMPARI1 and CaMPARI2, respectively) show the median RGR across all cells within a single mouse in a single light dose recording. Data were acquired from 6 CaMPARI1 mice recorded 1–3 times with different light doses (185–2,759 cells/mice and median value of 470 cells/mice) and from 11 CaMPARI2 mice recorded 1–3 times with different light doses (145–1,504 cells/mice and median value of 526 cells/mice). Data were fit with a linear model with zero intercept [black and blue solid lines for CaMPARI1 and CaMPARI2, respectively; slopes of 7.9 × 10^–4^ and 1.6 × 10^–4^ (RGR/light dose) for CaMPARI1 and CaMPARI2 and *p* = 3 × 10^–7^ and *p* = 2.6 × 10^–16^ for the model fit vs. null hypothesis, respectively; two-tailed *t*-tests]. Dashed lines show 95% confidence intervals. **(G)** Same representation as in **(F)** for excitation with 1,040 nm [slopes of 8 × 10^–3^ and 1 × 10^–3^ (RGR/light dose) for CaMPARI1 and CaMPARI2 and *p* = 0.0003 and *p* = 2 × 10^–11^ for the model fit vs. null hypothesis for CaMPARI1 and CaMPARI2, respectively; two-tailed *t*-tests; data from 4 CaMPARI1 mice with 443, 234, 185, and 571 cells/mouse; same CaMPARI2 mice as in **(F)**]. **(H)** No apparent change in CaMPARI2 PC efficiency was identified when it was expressed using three different AAV serotypes. Same CaMPARI2 data as in **(F)**, where squares show data from AAV1-CaMPARI2, circles show AAV5-CaMPARI2 data, and diamonds show AAV9-CaMPARI2 data. Within each serotype, data from different mice are shown in different colors (6 AAV1, 4 AAV5, and one AAV9 mice; mice were recorded 1–3 times using different light doses). The dashed line shows the model fit from **(F)**. **(I)** Simultaneous measurement of the activity levels of V1 and S1 neurons during the presentation of a drifting grating movie to the contralateral eye yielded higher activity levels in V1 neurons. This increase was significant when both CaMPARI1 and CaMPARI2 were expressed, as well as when 1,000 or 1,040 nm were used to read the RGR (data from *n* = 3 and 5 mice expressing CaMPARI1 and CaMPARI2, respectively). Each mouse was recorded 1–2 times using 1,000 or 1,040 nm and data are shown by triangles and squares, respectively; a light dose of ∼208 Joule/mm^2^ was used for all experiments; median values across all S1 and V1 neurons from the same mouse are connected with a line. 176–1,237 cells/brain region were recorded with median number of 423; *p* = 0.004 for comparing median RGRs across all V1 and S1 neurons for CaMPARI1-expressing neurons, and *p* = 0.007 for CaMPARI2-expressing neurons; two-tailed *t*-test). In addition, both S1 and V1 median RGRs where significantly higher when recorded with CaMPARI1 vs. CaMPARI2 (*p* = 0.007 and 0.022, respectively; Wilcoxon Ranksum Test). **(J)** Sensitivity index (d’) values for separating the activity of V1 and S1 neurons were similar when recorded with CaMPARI1 and CaMPARI2, as well as when 1,000 and 1,040 nm were used to read the RGR same data as in **(I)**. Mice were recorded using only 1,040 nm light (single wavelength, square dots) or using both 1,000 and 1,040 nm (dual wavelength, circular dots connected with a line). *p < 0.05, ^**^p < 0.01, and ^***^p < 0.001 for two-tailed *t*-tests; ^&^*p* < 0.05, ^&&^*p* < 0.01, and ^&&&^*p* < 0.001 for Wilcoxon Ranksum Tests.

For V1 PC experiments (shown in Figures 1, 2B–H), we modified the procedure used in previously published CaMPARI protocols (Fosque et al., 2015; Moeyaert et al., 2018). Mice were anesthetized using isoflurane (3% for induction and 1.5% during surgery) and placed on a heating pad. The heads of all mice were shaved, and a depilatory agent was briefly applied to render the skin bare. A skin incision was made and the locations of the primary visual cortices (V1) in both hemispheres were marked [2.8 mm lateral and 0.2 mm anterior to Lambda; injection coordinates were chosen according to the mouse brain atlas (Paxinos and Franklin, 2019)]. A 0.5 mm-diameter hole was drilled (Omnidrill35, World Precision Instruments) into each V1 location, and Adeno-associated virus (AAV) solution expressing either CaMPARI1 or CaMPARI2 under the human *synapsin* promoter, prepared by the Canadian Optogenetics and Vectorology Foundry viral vector core (RRID:SCR_016477) using Addgene plasmids #100832 and #101060, respectively, was administered. Three different AAV serotypes were used to compare CaMPARI1 and CaMPARI2 performance across different virus preparations: AAV1, AAV5, and AAV9 (2.2 × 10^13^, 2.7 × 10^13^, and 3.2 × 10^13^ GC/ml for CaMPARI1 AAV1, AAV5, and AAV9 solutions, respectively; 4 × 10^12^, 7 × 10^12^, and 1.7 × 10^13^ GC/ml for CaMPARI2 AAV1, AAV5, and AAV9 solutions, respectively). 100 nl of AAV solution were injected using a pulled and beveled glass micropipette (P-1000 and BV-10, respectively, Sutter Instruments; 50 nl/min injection rate) or a thin metal needle (34G, needle point style 4, Hamilton) into each V1 at depths of 200, 400, and 700 μm using an automated injection pump (Micro-2T, WPI). The needle was slowly and carefully withdrawn, with a 5-min wait period after each injection to allow for AAV diffusion into the brain. The skull openings were then sealed with bone wax (Johnson and Johnson Medical) and the surgical incision was closed with VetBond (3M). Following a recovery period of 3–4 weeks, mice were prepared for a second surgery. Mice were anesthetized as described above and a circular 2.5 mm diameter piece of bone was drilled around the center of each injection site. Cortex buffer (Holtmaat et al., 2012) was used consistently to keep the brain wet during the time of surgery and injections. The exposed brain was covered with a thin layer of low-melting point agarose (1% weight, IBI Scientific) and a 3 mm round glass coverslip (Warner Instruments) was gently pushed onto the brain surface. The glass was glued using a dental cement (Contemporary Ortho-Jet, Lang Dental), and the same cement was used to hold a custom-made metal headbar. Mice were then injected with chlorprothixene hydrochloride (20 μL of 0.33 mg/mL solution, intramuscular, Santa Cruz) and were given 30 min before imaging experiments were initiated.

**FIGURE 3:**
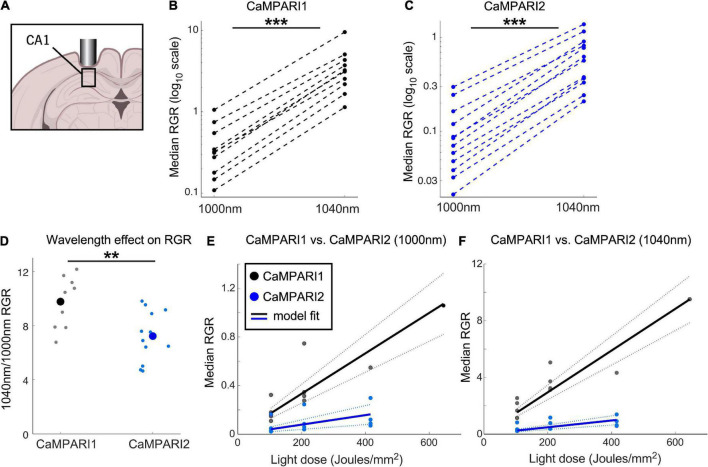
Comparing the PC performance of CaMPARI1 and CaMPARI2 in the mouse hippocampus. **(A)** Schematic illustration of hippocampal recording of CA1 neurons. **(B,C)** Comparing the median RGR for CaMPARI1 **(B)** and CaMPARI2 **(C)** shows similar increases in RGR with longer wavelength excitation to cortical recordings **(Figure 2)**. RGR was recorded from all identified cells in CA1 for all recording experiments and median values are shown (4 mice for CaMPARI1 with 370, 320, 461, and 258 cells/mouse; 3 mice for CaMPARI2, one mouse was recorded twice with 205 and 448 cells/recording and the other mice with 228, and 174 cells/mouse). The RGR readout was performed sequentially using 1,000 nm and then 1,040 nm excitation wavelengths. Lines connect data points acquired from the same mouse during the same session. Note the logarithmic scale of the y-axis. **(D)** The ratios of median RGR values acquired from the same mice at 1,000 and 1,040 nm excitation wavelengths were significantly higher for CaMPARI1 than CAMPARI2 mice [9.77 ± 1.82 vs. 7.22 ± 1.85, respectively, mean ± std.; *p* = 0.0062, Wilcoxon Ranksum Test; data from the same mice as in **(B,C)**]. Small circles show the ratio for the medians of individual recordings and the large circles are the means across all data points. **(E)** The median RGR across all recorded CA1 neurons showed linear increases with the light dose used for PC, with substantially higher PC rate for CaMPARI1 than CaMPARI2. The individual data points (gray and blue circles for CaMPARI1 and CaMPARI2, respectively) show the median RGR across all cells within a single mouse in a single light dose recording. Same mice as in **(B,C)**, each mouse was recorded 1–3 times with different light doses. Data were fit with a linear model with zero intercept [black and blue solid lines for CaMPARI1 and CaMPARI2; slopes of 1.7 × 10^–3^ and 3.9 × 10^–4^ (RGR/light dose) for CaMPARI1 and CaMPARI2, and *p* = 5 × 10^–6^ and *p* = 0.0011 for the model fit vs. null hypothesis for CaMPARI1 and CaMPARI2, respectively; two-tailed *t*-tests; same mice as in **(B)]**. Dashed lines show 95% confidence intervals. **(F)** Same representation as in **(E)** for excitation with 1,040 nm [slopes of 1.5 × 10^–2^ and 2.4 × 10^–3^ (RGR/light dose) for CaMPARI1 and CaMPARI2, and *p* = 4 × 10^–7^ and *p* = 4.5 × 10^–5^ for the model fit vs. null hypothesis for CaMPARI1 and CaMPARI2, respectively; two-tailed *t*-tests; same mice as in **(C,E)]**. **p < 0.01, ***p < 0.001.

For hippocampal PC experiments (shown in Figure 3), we followed previously published procedures (Dombeck et al., 2010; Das et al., 2020). We anesthetized the mice as described above, injected local pain medication (Bupivacaine 0.5%), and drilled out a 3-mm diameter piece of the skull centered around 2 mm lateral and 2.2 mm caudal to Bregma. The cortical tissue and corpus callosum were gently aspirated away by using sharp and blunt 28G needles connected to a vacuum line to reveal the hippocampal surface. Cortex buffer was used consistently to keep the brain wet during the time of surgery and injections. AAV1 solution of either CaMPARI1 or CaMPARI2 (same AAVs as above) was injected into three adjacent locations forming a triangular shape with 200–300 μm distance between sites. In each penetration site, 100 nL AAV solution was injected (Micro-2T, WPI) at depths of 200 and 400 μm under the exposed hippocampal surface. A round 3 mm diameter glass coverslip (Warner Instruments) glued to the bottom of a metal cannula (type 304L, New England Small Tube Corporation) was pushed gently into place and secured using glue (Krazy glue) and dental cement (Contemporary Ortho-Jet), which were also used to secure a custom-made metal headbar to the mouse skull. Mice were given a minimum time of 3 weeks post-injection to allow for recovery. Before recording of hippocampal activity, mice were injected with chlorprothixene hydrochloride (30 μL of 0.33 mg/mL solution, intramuscular) 30 min before the recording session started.

**FIGURE 4:**
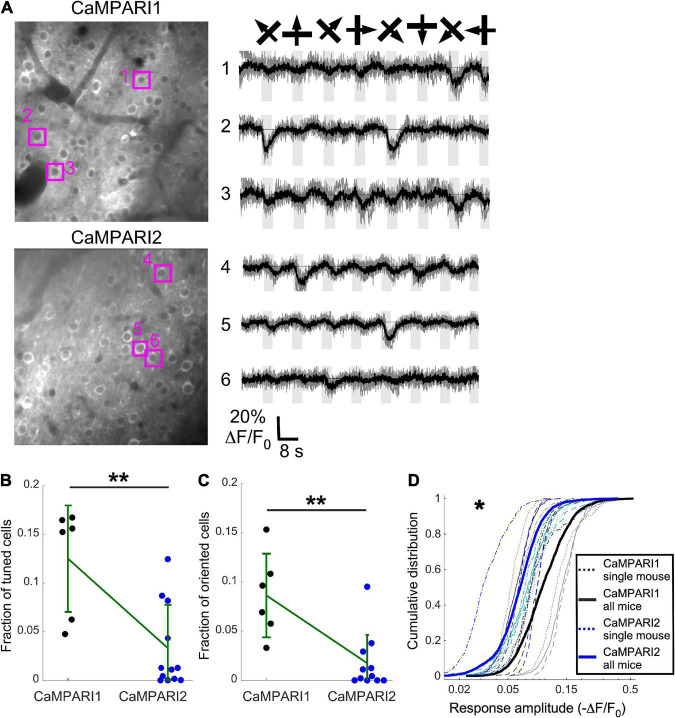
CaMPARI1 outperforms CaMPARI2 for recording dynamic activity patterns in the mouse V1. **(A)** Example images (left) and example traces (right) of CaMPARI1- (top) and CaMPARI2-expressing (bottom) neurons in the mouse V1. The example traces were selected from tuned and oriented cells, and the respective cell locations are highlighted on the image. **(B,C)** The fractions of tuned **(B)** and oriented **(C)** neurons in V1 were higher for CaMPARI1-expressing than CaMAPRI2-expressing mice [*n* = 4 CaMPARI1 mice, with two mice recorded twice (*n* = 502, 692, 208, 498, 433, and 757 neurons/recording); *n* = 8 CaMPARI2 mice, with three mice recorded twice (122–942 cells/recording with median number of 273)]. The increase was significant for the fraction of tuned cells (0.125 ± 0.055 vs. 0.033 ± 0.045 for mean ± standard deviation of CaMPARI1 vs. CaMPARI2, respectively; *p* = 0.005, Wilcoxon Ranksum Test) as wells as the fraction of oriented cells (0.086 ± 0.043 vs. 0.018 ± 0.029; *p* = 0.002, Wilcoxon Ranksum Test). Error bars show the standard deviation for each sensor and the mean values are connected with a green line. **(D)** The cumulative distribution of the peak response amplitude for a visual stimulus of all recorded V1 neurons from all mice shows a significant decrease in CaMPARI1’s response amplitudes [median value across all median cumulative distributions of –0.105 ± 0.035 for CaMPARI1 vs. –0.072 ± 0.016 for CAMPARI2; median ± standard deviation; *p* = 0.02; Wilcoxon Ranksum Test between the median values of all recorded neurons on the same recording session; thin dashed lines show data from single recordings and solid lines show the piled distribution from all recorded mice with the same sensor; same mice as in **(B,C)]**. *p < 0.05, **p < 0.01.

For dynamic recording from V1 neurons (Figure 4), mice were anesthetized using isoflurane (3% for induction, 1.5% during the surgery) and placed on a heating pad. Each mouse was injected with local pain medication (bupivacaine 0.5%) and the skull bone above the left cortical hemisphere was exposed. A circular 3 mm diameter craniotomy was drilled (Omnidrill35, World Precision Instruments) over an area covering V1 (centered around –2.8 mm lateral and +0.2 mm from Lambda). AAV solution expressing either the CaMPARI1 or CaMPARI2 sensors (both AAV1s as injected above from the Canadian Optogenetics and Vectorology Foundry viral vector core, as well as AAV1-CaMPARI1 and AAV1-CaMPARI2, catalog numbers 00832-AAV1 and 101060-AAV1, respectively, from Addgene) were injected into two locations, separated by ∼300 μm, into each cortical region (3 injection depths per location, 200, 400, and 700 μm under the pia; 50 nL AAV solution in each depth) using an automated injection pump (Fusion 200 touch Syringe Pump, Chemyx, and Micro-2T, WPI) and a pulled and beveled micropipette (P-1000 and BV-10, respectively, Sutter Instruments). Cortex buffer was used consistently to keep the brain wet during the time of surgery and injections. Following the viral injection, a cranial window (two glued layers of circular glass, Warner Instruments) was placed carefully, and a custom-made metallic head bar was attached using dental cement (Contemporary Ortho-Jet). Animals were injected with Buprenorphine (0.1 mg/kg) and Ketoprofen (5 mg/kg, immediately, 24, and 48 h after the surgery) for post-operative care and animals were allowed a minimal recovery time of 3 weeks before the start of experiments. For comparing V1 and primary somatosensory cortical (S1) activity (shown in Figures 2I–J; these mice were also used to record dynamic activity of V1 neurons), a similar surgery was conducted, where a 3 × 5 mm^2^ craniotomy was drilled to cover the area from Bregma to Lambda and from –0.5 to –3.5 mm lateral to midline. Injection coordinates were chosen according to the mouse brain atlas (Paxinos and Franklin, 2019): 2.8 lateral and 0.2 mm anterior to Lambda (V1), and 2.5 mm lateral and 3.4 anterior to Lambda (S1), and the same AAVs were injected into V1 and S1. The craniotomy was covered with a custom 3 × 5 mm^2^ glass window (Tower Optical), and animals were given 3–4 weeks for recovery before imaging started.

### Recording of neuronal activity

For recording V1 activity using PC, lightly anesthetized and sedated mice (0.5% isoflurane and injected with Chlorprothixene Hydrochloride) were moved from the surgery rig and were head-fixed under a multiphoton microscope (Bergamo II, Thorlabs) controlled by ThorImage software. The microscope arm was rotated (typically 10–15° laterally) to reach to a perpendicular angle on top of each hemispheric window. A laser source (Insight X3, Spectra Physics) was used to excite fluorescence at 1,000 and 1,040 nm. The microscope was equipped with galvo/resonant scanners, two GaAsP PMT detectors for green- and red-channel detection with 525/50 and 607/70 nm filters, respectively, separated by a 565 nm dichroic filter (all from Chroma). PC was generated using a broadband light source (X-Cite Fire lamp, Excelitas) and a 400/40 nm bandpass filter (Brightline, Semrock).

For all mice, we recorded the CaMPARI signal before PC (pre-PC data), which was used to compare the green signal brightness across the different CaMPARIs. For both PC and dynamic activity recording experiments, mice were presented with a visual stimulus consisted of a drifting sinusoidal grating moving in 8 directions for 4 s with 1 Hz temporal frequency [Psychophysical Toolbox (Brainard, 1997; Pelli, 1997)], followed by 4–12 s of gray display (12 s for PC experiments, and either 4 or 8 s for dynamic recording experiments). The stimulus was presented on an LCD monitor covered with a blue Plexiglass filter to minimize light penetration to the green and red fluorescence channels (30 ×36 cm^2^ display, located 15 cm in from of the mouse eye, and tilted 45 degrees in respect to the nose line) that subtended an angle of ± 50° horizontally and ± 45° vertically around the mouse’s eye. The stimulation cycle was repeated 5 times. The PC light was delivered for 1 s during the presentation of each drifting grating, 1.5 s after the grating appeared, and the no-stimulation period after it was designed to allow the tissue to cool down. The PC light covered a ∼7 mm diameter circle around the cranial window (but did not illuminate the contralateral hemispheric cranial window) and the power was set to 100–200 mW on the sample plane. After the visual stimulation was completed, we recorded the red and green CaMPARI somatic signals. Then, an additional 1–2 cycles of PC and recording were conducted for most of the mice, testing different combinations of PC light intensities and illumination times to calculate the dependence of the red-to-green ratio (RGR) upon the PC light dose. Readout of the RGR was conducted by two-photon microscopy of all identified neurons within a ∼700 × 700 × 250 μm^3^ volume in V1, using 1,000 and 1,040 nm excitation light, 1,024 × 1,024 pixels/image, and a 10x, 0.5NA objective (TL10X-2P, Thorlabs). Multiple recordings were conducted with several mice, as we have previously shown that CaMPARI’s red signal decays and allow new recordings ∼7 days after the initial PC (Das et al., 2022). Repeating recordings were conducted using the same experimental conditions as the first recordings, and at least 7 days after the initial recording. Dynamic recording was conducted by recording the green CaMPARI fluorescence using 950 nm excitation light. The field of view (FOV) size was ∼300 × 300 μm^2^, with 512 × 512 pixels acquired at 30 frames per second, and using a 16x, 0.8NA objective (CFI75 LWD 16X W, Nikon).

### Data analysis

Cell segmentation for PC recording was performed using CellPose (automatic segmentation) (Stringer et al., 2021) and custom MATLAB (Mathworks) scripts. Following Cellpose segmentation of cells within the raw images, data were curated by an experimenter, and then imported into Matlab. The dark current, defined as the 0.2 percentile of the recorded data for each color channel, was subtracted from the raw data. Since CaMPARI’s green signal penetrates the red channel (Fosque et al., 2015), we calculated and corrected for the red-to-green contamination ratio by recording CaMPARI images before PC (pre-PC) for both 1,000 and 1,040 nm excitation and quantified the contamination ratio. Green-to-red correction values were 13.1 and 14.6% for the for 1,000 and 1,040 nm data, respectively, which were also subtracted from the red channel. Following these corrections, we calculated the post-PC RGR (defined as the ratio of fluorescence in the red channel with 607/70 nm filter, to the fluorescence in the green channel with 525/50 nm filter) for all identified cells and used the median value for comparisons across animals. We note that since CaMPARI’s PC occurs in all CaMPARI-expressing neurons simultaneously, the RGR was piled from all cells across all recorded fields of view and the median was calculated from this population of cells. We also calculated the sensitivity index (d’) to measure the separation between all cells recorded in V1 and S1 regions using the following formula:


$$d^{\prime }=\frac{\left(mean_{RGR_{visual}}-mean_{RGR_{somatosensory}}\right)}{\surd (0.5\left(variance_{RGR_{visual}}+variance_{RGR_{somatosensory}}\right)}$$

For light dose calculations, we consecutively photoconverted the same brain region 1–3 times on the same day and measured the RGR from all cells after each PC. The light dose was calculated as the total light intensity (100 or 200 mW) for each PC event divided by the illumination cross-section (a 7 mm-diameter circle) and multiplied by the illumination time (1 or 2 s).

For calculating the dynamic changes in CaMPARI fluorescence, we followed a similar approach to previously published CaMPARI and other GECI works (Fosque et al., 2015; Dana et al., 2016; Moeyaert et al., 2018; Dana et al., 2019; Das et al., 2022). Small drifts and movements of the imaged area throughout recording were corrected using TurboReg plug-in of ImageJ (Thevenaz et al., 1998). Fluorescence bleaching was corrected for each cell using 7-th order polynomial fit, and cell bodies were segmented within each FOV using a semi-automated algorithm (Chen et al., 2013) and extracted the cellular signal. We calculated the baseline fluorescence (F_base_) during the 0.66 s before the appearance of each grating moving in any direction, and the response fluorescence (F_resp_) as the average over the lowest 25% of fluorescence values during the 4 s each grating direction was presented. The fluorescence change (ΔF/F_0_) was calculated as ΔF/F_0_ = (F_resp_-F_base_)/F_base_. We note that since CaMPARI is a negative GECI, these values were negative. For each cell, we conducted an ANOVA test among the 80 F_base_ and F_resp_ values for each recorded movie (8 grating movement directions × 5 repetitions). Cells with *p* < 0.01 were classified as “tuned cells.” For the tuned cells, we conducted an additional ANOVA test among the 40 F_resp_ values to find significant changes among the different directions. Cells with *p* < 0.01 were also classified as “oriented cells.”

## Results

### Side-by-side comparison of CaMPARI1 and CaMPARI2 in the mouse V1

Previous studies characterized the PC and dynamic recording properties of CaMPARI1 and CaMPARI2 (Fosque et al., 2015; Moeyaert et al., 2018), but there was no reported direct *in vivo* comparison among their performance in the mouse brain. To directly compare these sensors, we injected 8–16-week-old mice with AAVs expressing the CaMPARI1 and CaMPARI2 sequences into the V1 region and implanted them with cranial windows above the injection site (see section “Materials and methods”). After 3–4 weeks, we recorded neuronal activity from layer II/III neurons in V1 of lightly anesthetized mice presented with drifting grating movies (Methods). As expected, green CaMPARI fluorescence was evident in neurons before PC, and red signal was detectable after PC (Figure 1A). We compared the readout of CaMPARI’s fluorescence using two excitation wavelengths, 1,000 and 1,040 nm, as were respectively, used for the CaMPARI1 and CaMPARI2 testing (Fosque et al., 2015; Moeyaert et al., 2018). As expected, 1,040 nm illumination led to more efficient excitation of the red component and less efficient excitation of the green component, and therefore to higher RGR compared to 1,000 nm illumination (Figure 1B).

Switching to a longer wavelength excitation, and the enhanced RGR associated with it, may assist in increasing the sensitivity of the sensor readout. We photoconverted the V1 of CaMPARI1- and CaMPARI2-expressing mice [*n* = 6, (3 males and 3 females), and *n* = 11, (3 males and 8 females) mice, respectively, with 1–3 consecutive PC recordings from each mouse; Figure 2A] during visual stimulation and recorded the red and green CaMPARI fluorescence from all identified cells (see section “Materials and methods”). We found that the increase in RGR with longer illumination wavelength was significant and generally maintained the same rank order of the neuronal PCs (Figures 2B, C; *p* = 0.021 and 6 × 10^–8^ for CaMPARI1 and CaMPARI2, respectively; paired two-tailed *t*-test). The median RGR increase was significantly higher for CaMPARI1 than CaMPARI2, with 9.05- vs. 5.8-fold increase, respectively (*p* = 0.0009, Wilcoxon Ranksum Test; Figure 2D), which presumably resulted from small differences in the excitation and emission spectra of the two constructs. Notably, the green fluorescence of CaMPARI2 was significantly brighter than that of CaMPARI1, in agreement with the previous *in vitro* characterization (Moeyaert et al., 2018; Figure 2E).

Next, we compared the PC rate of the two constructs for different PC light doses by illuminating quanta of ∼100 Joules/mm^2^ onto the mouse cranial window, which were synchronized with the presentation of a drifting grating movie to the contralateral eye. Since CaMPARI’s PC process was shown to be relatively linear with the expected range of firing rate (Fosque et al., 2015; Moeyaert et al., 2018), we conducted up to 3 PC cycles for each mouse and measured the RGR after each cycle. For all recorded mice, RGR for CaMPARI1 was substantially larger than for CaMPARI2. The results were fit with a linear regression model with zero intercept for both datasets excited with 1,000 and 1,040 nm. The slope of the CaMPARI1 models, which quantifies the amount of RGR change per unit of PC light, was 4.85- and 7.8- fold larger than for the CaMPARI2 models (Figures 2F, G; *P* < 0.0003 for *t*-tests of all slope fits vs. null hypothesis), indicating that CaMPARI2 suffers from reduced PC efficiency under these experimental conditions. To check the option that impaired CaMPARI2 performance was due to an inefficient viral expression, we used three different AAV serotypes to express CaMPARI2: AAV1, AAV5, and AAV9. CaMPARI2 performance was similar for all three AAVs (6 mice expressing AAV1-CaMPARI2, 4 mice expressing AAV5-CaMPARI2, and one mouse expressing AAV9-CaMPARI2: Figure 2H).

One of the reported advantages of CaMPARI2 over CaMPARI1 is its reduced background PC, i.e., the PC that occurs under low Ca^2+^ concentrations (Moeyaert et al., 2018). We tested this property by expressing either CaMPARI1 or CaMPARI2 in both V1 and the primary somatosensory cortex (S1) of mice [*n* = 3 (1 male and 2 females) and *n* = 5 (3 males and 2 females) mice, respectively; 1–3 recordings from each mouse, see section “Materials and methods”]. We photoconverted both brain regions while presenting the mice with a drifting grating movie to their contralateral eye. Then, we measured RGR from all V1 and S1 neurons. We compared the PC level in the non-directly stimulated S1 neurons, the stimulated V1 neurons, and also calculated the sensitivity index (d’, see section “Materials and methods”) for separating the activity levels in both of these brain regions (Das et al., 2022). We found that, as expected by the reported *in vitro* data, CaMPARI2 baseline PC in S1 was lower than that of CaMPARI1. The mean RGR for S1 neurons excited with 1,040 nm light was ∼35% of that of CaMPARI1 for the same light dose (*p* = 0.029, Wilcoxon Ranksum Test; Figure 2I), but this advantage was balanced by a ∼300% increase in CaMPARI1 RGR in V1 compared to CaMPARI2 (*p* = 0.004, Wilcoxon Ranksum Test, Figure 2I). When calculating the sensitivity index for RGR acquired with 1,040 nm excitation, CaMPARI1 showed a small, non-significant increase in d’ over CaMPARI2 (2.26 ± 0.89 vs. 1.95 ± 0.71, respectively; mean ± std; *p* = 0.6, Wilcoxon Ranksum Test; Figure 2J). Moreover, comparing d’ values between experiments with 1,000 or 1,040 nm excitation wavelength showed no significant advantage for using either wavelength (Figure 2J).

### Side-by-side comparison of CaMPARI1 and CaMPARI2 in mouse CA1 hippocampal neurons

To extend CaMPARI characterization to test the sensors’ performance in non-cortical neurons, we injected mice with AAVs expressing CaMPARI1 and CaMPARI2, and implanted them with hippocampal windows (Figure 3A; *n* = 4 and 3 male mice for CaMPARI1 and CaMPARI2, respectively; 8–16 weeks old; see section “Materials and methods”). Following a minimum recovery time of 4 weeks, we recorded spontaneous neuronal activity by illuminating the hippocampal window with PC light while the lightly anesthetized mice were kept on a heating pad in the dark. Previous experiments under similar conditions with the more sensitive GECI jRGECO1a (Dana et al., 2016, 2018; Das et al., 2020) showed that most of the CA1 excitatory neurons showed relatively high spontaneous activity rates, making CA1 recording an attractive option for further characterization of CaMPARI performance in subcortical regions.

Similar to the stimulus-evoked PC in the cortex, we identified a significant increase in RGR when 1,040 nm excitation light was used to measure the RGR vs. 1,000 nm light (Figures 3B, C, P = 0.0007 and 0.00005 for CaMPARI1 and CaMPARI2, respectively; paired two-tailed *t*-tests). The median increase was slightly higher than in cortical neurons, with 9.77- and 7.22-fold increases for CaMPARI1 and CaMPARI2, respectively, with significant differences among the sensors (Figure 3D *p* = 0.006, Wilcoxon Ranksum Test). Testing the dependence of RGR levels on PC light dose also yielded that CaMPARI1 sensitivity outperformed CaMPARI2, with 4.25- and 6.15- fold larger model-fit slopes for 1,000 and 1,040 nm excitation, respectively (Figures 3E, F; *P* < 0.01 for *t*-tests of all slope fits vs. null hypothesis). Interestingly, comparing the model-fit slopes for the same constructs and readout wavelengths across V1 and CA1 regions showed 1.82–2.41-fold increases for the CA1 region, indicating larger overall activity levels in CA1 neurons under these experimental conditions (*p* = 0.0006, *t*-test for the ratio of slope coefficient; Figures 2F, G, 3E, F).

### Side-by-side comparison of dynamic recording with CaMPARI1 and CaMPARI2 in the mouse V1

Although CaMPARI’s unique characteristic is its PC capability, it can also be used as a regular GECI to monitor dynamic changes in neuronal activity (Fosque et al., 2015; Moeyaert et al., 2018). We compared CaMPARI1 and CaMPARI2 by injecting 8–16-week-old mice with AAV expressing CaMPARI1 and CaMPARI2 sequences into their V1 [*n* = 4 (2 males and 2 females) and *n* = 8 (2 males and 6 females) mice, respectively, with 1–2 recordings from each mouse]. After 3–4 weeks of recovery, the mice were lightly anesthetized, sedated, and placed in the dark on a heating pad. A drifting grating movie was presented to their contralateral eye, and we recorded the green CaMPARI fluorescence signal using 950 nm excitation light (see Methods). In agreement with previously reported data (Fosque et al., 2015; Moeyaert et al., 2018), we found that both CaMPARI sensors exhibited fluorescence decreases during the presentation of a visual stimulus in some of the recorded neurons. We quantified the fraction of neurons in each recorded FOV that showed significantly reduced fluorescence during visual stimulation vs. periods with no stimulation (tuned cells; ANOVA test with *p* < 0.01, see Methods), as well as neurons that showed significantly reduced fluorescence during the presentation of one or more grating orientations (oriented cells; ANOVA test with *p* < 0.01, Figure 4A; see section “Materials and methods”). Fractions of both tuned and oriented cells were higher for CaMPARI1-expressing mice (Figures 4B, C *p* = 0.005 and *p* = 0.002 for tuned and oriented cells, respectively; Wilcoxon Ranksum Test). In addition, the measured response amplitudes (absolute value of ΔF/F_0_, see “Materials and methods”) were significantly higher for CaMPARI1-expressing neurons (Figure 4D; *p* = 0.02, Wilcoxon Ranksum Test for comparing median amplitudes from each mouse’s cumulative distribution). These findings indicate that CaMPARI1 is also a more sensitive sensor than CaMPARI2 for detecting dynamic changes in stimulated V1 activity.

## Discussion

In this study, CaMPARI1 and CaMPARI2 were compared under several experimental conditions. CaMPARI’s PC rate was measured in the living mouse cortex and hippocampus for recording stimulated and spontaneous activity patterns using various light doses. The dynamic recording capabilities of the sensors were compared for detecting stimulated activity in V1 neurons. In addition, CaMPARI was used to simultaneously record from two cortical regions, V1 and S1, during visual stimulation, and to compare their activity levels. The latter experiment allows exploiting a major advantage of CaMPARI over other calcium sensors, which is its ability to simultaneously record volumetric, single-cell activity patterns across multiple brain regions (Das et al., 2022). For this experimental paradigm, S1 serves as a same-subject reference point for a brain region that is adjacent to the visual cortex, but does not receive substantial direct input from the visual pathway. In agreement with this approach, the measured activity level of V1 neurons was higher than S1 neurons for all recorded mice using both sensors (Figure 2I).

Direct comparison of CaMPARI1 and CaPARI2 performance in the mouse V1, S1, and CA1 neurons using two different wavelengths (Figures 2B, C, 3B, C) yielded surprising results. Although CaMPARI2 showed brighter green fluorescence signal, reduced background PC in S1 neurons, and similar ability to quantify the difference between V1 and S1 neurons, its overall *in vivo* performance was inferior to CaMPARI1. We identified a significant decrease of 5–10-fold in CaMPARI2 PC rate compared to CaMPARI1 (Figures 2F, G, 3E, F), which was consistent for the two excitation wavelengths that were used. This reduction was unexpected based upon the reported *in vitro* characterization results (Moeyaert et al., 2018) and highlights a potential limitation of this screening pipeline’s predictive power. In addition, the dynamic recording sensitivity of CaMPARI2 was also reduced compared to CaMPARI1 (Figure 4), making CaMPARI1 the sensor of choice for the tested experimental conditions. We note that the CaMPARI2 Ca^2+^ affinity is lower than that of CaMPARI1 [K_d_ of 287 vs. 111 nM, respectively (Fosque et al., 2015; Moeyaert et al., 2018)]. However, when a higher affinity variant of CaMPARI2 (CaMPARI2-F391W) was previously tested in the mouse V1, it showed reduced specificity in its labeling of cells based upon their activity (Moeyaert et al., 2018). Therefore, the reduced performance level of CaMPARI2 cannot be solely attributed to its lower affinity.

One possible way to increase the measured CaMPARI RGR *in vivo* is to read the photoconverted signal using a longer excitation wavelength, which more effectively excites the red component and reduces the green component’s excitation efficiency. Based on the excitation spectra of CaMPARI1 and CaMPARI2 (Fosque et al., 2015; Moeyaert et al., 2018), we increased the excitation wavelength from 1,000 to 1,040 nm, which is close to the edge of the CaMPARI green excitation spectrum. Under these conditions, we detected a larger increase in CaMPARI1’s RGR over CaMPARI2, which further increased the difference between the two sensors (Figures 2D, 3D) but did not significantly change the sensitivity index values for detecting differences across V1 and S1 activity (Figure 2J). Interestingly, CaMPARI2’s PC rate using 1,040 nm excitation was similar (∼10–40% higher) to that of CaMPARI1 at 1,000 nm excitation [slopes of 8 × 10^–4^ and 1.7 × 10^–3^ (RGR/light dose) for cortical and hippocampal recording with CaMPARI1 at 1,000 nm, respectively; and 8.8 × 10^–4^ and 2.4 × 10^–3^ (RGR/light dose unit) for cortical and hippocampal recording with CaMPARI2 at 1,040 nm, respectively], similarly to the reported nominal RGR levels when CaMPARI1 and CaMPARI2 were tested *in vivo* using 1,000 and 1,040 nm, respectively (Fosque et al., 2015; Moeyaert et al., 2018). Therefore, switching to a longer wavelength is a good strategy to increase the recorded RGR, specifically if the green signal is bright and the red is dim, but it does not provide superior performance level under the tested conditions, and it does not eliminate the performance gap between the two sensors.

Optimizing the performance of GECIs and other protein sensors is a critical step for transforming a promising concept into a widely used tool. For example, the optimization of the first-generation GCaMP GECI has continued for more than 20 years, with gradual increases in the number of screened constructs for the newer generations of the sensor, and with the recent jGCaMP8, X-CaMPG, and G-CaMP9a descendants showing superior performance compared to earlier generations (Nakai et al., 2001; Inoue et al., 2019; Zhang et al., 2021; Sakamoto et al., 2022). Switching to large-scale, *in-vitro*-based optimization pipelines is not limited to GECIs, but was also demonstrated for improving the performance of genetically encoded voltage sensors (Kannan et al., 2018, 2021) and glutamate sensors (Aggarwal et al., 2022). To enhance the predictivity of these screening assays, previous works have incorporated screening with cell types that are similar to the target applications, such as cultured mammalian neurons (Chen et al., 2013; Dana et al., 2016, 2019; Zhang et al., 2021). The development of CaMPARI2 used this state-of-the-art optimization approach, with multiple steps of mutagenesis and testing *in vitro* and proof-of-concept experiments *in vivo* (Fosque et al., 2015; Moeyaert et al., 2018). However, our work in this study shows that following this *in vitro*-based screening pipeline may still yield inaccurate predictions of *in vivo* performance. Notably, *in vitro* screening is an essential step, as it allows high-throughput characterization of important sensor parameters such as affinity, Hill coefficient, K_on_ and K_off_, and excitation and emission spectra. Such in-depth characterization is challenging using *in vivo* assays. However, the presented findings suggest that functional parameters, such as PC rate in stimulated and non-stimulated cells, which may be characterized using *in vivo* assays, may deviate from the *in vitro* predictions. Therefore, we suggest that future screening pipelines should incorporate an additional *in vivo* screening step to characterize the functional properties of sensors. We note that *in vivo* experimental throughput is typically substantially lower than what may be achieved *in vitro*, and therefore this additional step needs to be limited to a smaller number of carefully selected candidates. The identification of which sensor features may by reliably assessed by *in vitro* screening is thus central for reducing the rate-limiting *in vivo* screening and will require future works to generate comparative databases for defining such parameters, which may also differ among sensors.

## Data availability statement

The original contributions presented in this study are included in the article/supplementary material, further inquiries can be directed to the corresponding author.

## Ethics statement

This animal study was reviewed and approved by the Lerner Research Institute Animal Care and Use Committee (IACUC) and Institutional Biosafety Committee (IBC).

## Author contributions

HD and AD conceived the project and designed the study. AD and DM performed all surgeries. AD, DM, and JB performed the experiments. JI, JB, DP, AD, and DM analyzed the data. M-EP designed and provided the AAVs for this study. HD wrote the manuscript with comments from all authors. All authors contributed to the article and approved the submitted version.
